# Extrinsically Integrated Instructional Quizzes in Learning Games: An Educational Disaster or Not?

**DOI:** 10.3389/fpsyg.2021.678380

**Published:** 2021-08-19

**Authors:** Lucie Jičínská, Petra Sedláčková, Lukáš Kolek, Tereza Tetourová, Kristina Volná, Jiří Lukavský, Cyril Brom

**Affiliations:** ^1^Faculty of Education, Charles University, Prague, Czechia; ^2^Faculty of Mathematics and Physics, Charles University, Prague, Czechia; ^3^Development and New Media (decko.cz and ctart.cz), Czech Television, Prague, Czechia; ^4^Institute of Psychology, Czech Academy of Sciences, Prague, Czechia

**Keywords:** game-based learning, educational games, quiz, instructional quiz, multiple-choice, extrinsic integration, experiment, learning outcomes

## Abstract

Instructional quizzes are frequently used in educational games. When they present correct answers after learners have responded, these quizzes can be used on their own for teaching new factual and conceptual knowledge (no additional learning materials are needed). In games, these quizzes are often unrelated to gameplay: gameplay can be viewed as a reward for answering quiz questions. This has been criticized in game-based learning literature as a “chocolate-covered-broccoli” approach. However, is it really a bad approach? Theories offer conflicting predictions concerning the instructional efficiency of in-game quizzes relative to bare quizzes (i.e., not embedded in games) and empirical literature is lacking. Here, we present a within-subject design study (*N* = 69), in which 10–12-year-olds learn from both an in-game quiz and a bare quiz and undergo immediate and 2–3 weeks delayed post-test on the quiz questions. A modest difference in learning outcomes favoring the bare quiz was found in the immediate post-tests (*d* = 0.46), but not in the 2–3 weeks delayed post-tests (*d* = 0.09). Children enjoyed the game more than the bare quiz (*d*_*z*_ = 0.65) and 59 preferred the game in the free-choice period. The findings suggest that both a bare quiz and a quiz within a game have their place at the table for useful educational interventions: the bare quiz should be preferred in schooling contexts; whereas, the game in leisure time situations as a voluntary activity. In the latter case, it should be considered how the game and the quiz are integrated.

## Introduction

Quizzes are used in education for many reasons. For instance, they provide retrieval practice (Roediger et al., 2011; Rowland, 2014; Adesope et al., 2017; Sanchez et al., 2020), present performance feedback (Keough, 2012; Chien et al., 2016; Hunsu et al., 2016), assist in drill-and-practice exercises (Crompton et al., 2017; Hung et al., 2018), or—when used collectively in a classroom—improve interaction and attention levels (Hunsu et al., 2016). Quizzes are typically used as supplements to other types of instruction: after an initial period of studying, learners complete a quiz. Quizzing learners this way—be it individually or collectively, in a classroom or at home—generally improves learning outcomes (see Rowland, 2014; Chien et al., 2016; Hunsu et al., 2016; Adesope et al., 2017; Wang and Tahir, 2020).

Quizzes can also teach new factual and conceptual knowledge in and of themselves without additional instruction. This happens when the quiz provides feedback on the correctness of learners’ responses. When learners study from these *instructional quizzes*, they need no additional instructional materials—other than the quiz questions and the feedback. Instructional quizzes are often used in single-player educational games; for instance, in specific language-learning games (Hung et al., 2018). Such games can be played voluntarily in leisure time, yet they can also support the acquisition of new knowledge by quizzing the player. Instructional quizzes within a single-player educational game are the focus of this study.

Probably the most convenient way to integrate instructional quizzes into single-player games is presented by the *extrinsic integration* mechanic, as evidenced by a plethora of on-the-shelf edutainment software. Extrinsic integration means that gameplay is basically interrupted every few minutes by a quiz practically *unrelated* to the game. Once the quiz has been completed, the player may continue playing as a reward. This is the so-called “chocolate-covered broccoli” approach: making an “unpalatable” quiz (broccoli) more “palatable” by wrapping it in a game (chocolate). Drawing on motivational literature (Ryan and Deci, 2000), this approach has been widely criticized in the game-based learning community (e.g., Habgood and Ainsworth, 2011) because it interrupts game-induced flow. Plus, from the perspective of cognitive theories of learning (e.g., Mayer, 2009; Sweller et al., 2011), playing the game is a seductive detail that distracts the learner’s attention away from the quiz. Consequently, learning from a quiz within an unrelated game should be inferior to learning from a bare instructional quiz.

However, from a more behavioristic perspective, extrinsic rewards can actually increase motivation; especially, but not only, when given for relatively low-interest tasks (see Cameron et al., 2001), such as answering quiz questions. Recent gamification meta-analyses support the position that game elements extrinsically integrated with instruction can increase academic achievement (see Bai et al., 2020; Huang et al., 2020). This research tradition would argue that gamified quizzes, even when the quiz is unrelated to the gameplay, may be instructionally superior to their non-gamified counterparts.

Empirical evidence on the instructional efficiency of extrinsically integrated in-game quizzes is lacking. The aim of this study is to provide such evidence as concerns children in Grades 5–6. The results can help elucidate the limits of the theories above and, from a practical perspective, offer insights on whether or not to use in-game instructional quizzes.

## Methods

### Design and Dependent Variables

This lab study used a 2×2 within-subject design. The first factor was related to the order of interventions: participants were exposed, in a random order, both to a game including an instructional quiz and to a bare quiz. The second factor was related to the order of questions. We prepared two sets of quiz questions: each with twelve questions (Set A, Set B). The game showed one of the sets and the quiz the other; the order of sets was chosen randomly. So, the design was as follows: order game-first| quiz-first × set order AB| BA.

The study followed a pre-post-delayed test protocol. We used the score from quiz questions given during the intervention as the **pre-test** variable. The **post-test** was administered directly in the lab immediately after the intervention. In the lab, we also measured **perceived enjoyment** of interfacing with the game and bare quiz and free-choice **motivation**. These are two complementary, affective-motivational measures. Enjoyment is viewed here as a positive, activity-related, activating affective-motivational state (Pekrun, 2006) and motivation as a propensity to start, continue or stop performing a specific activity in the current context (Ryan and Deci, 2000). Finally, the **delayed test** was administered roughly 3 weeks later via phone.

### Participants

Sixty-nine Czech children (age: 10–12; 48% girls) participated. A power analysis for a within-subject design (game vs. bare quiz, expecting a medium effect size *d*_*z*_ = 0.5) suggested that we need at least 44 children for power 0.90. An additional three children were excluded due to technical errors. Participants were recruited via a website and through social network calls on Czech TV’s children’s channel. They received a LEGO kit or a board game worth 20 EUR for their participation.

Most children participated between July and September 2020 between the first and the second wave of COVID-19 in the Czech Republic. They all had just finished the fifth grade. They were accompanied to the lab by their parents. From there on, they continued individually in the experimental process (a few of them, such as those living in the same household, participated in small groups). Some participants wore masks during the experiment (of their own will). The staff wore masks.

There was no significant difference between participants in the four groups (order game-first| quiz-first × set order AB| BA) in terms of gender, prior mood, and domain interest in the topics addressed in the quiz questions (Supplementary Material A).

### Materials

#### Knowledge Questions

As stated above, we prepared 2×12 questions, each in two formats: a multiple-choice one and a short answer one. The multiple-choice format featured 4 possible answers for every question, one being the correct answer (scale for each question set: 0–12; chance level: 3 points). This format was used for instructional quizzes; i.e., in the game and in the bare quiz. Answers to the in-game and in-bare-quiz questions constituted the **pre-test**. The short answer format was utilized for the **post-test** given in the lab in written form and for the **delayed-test** administered via phone. For answering each short answer question, the participant could receive 0, 0.25, 0.5, 0.75, or 1 point (graded by two raters; disagreement resolved by consensus). The scale for each question set is, again, 0–12, but the chance level is 0.

Most of the questions were factual; some were conceptual. The questions’ topics included the following: space, the human body, nature, earth geology (Supplementary Material B lists the questions). The questions were piloted before the study started to verify that the answers are mostly unknown to fifth graders but can be learned by them (*n_pilot_* = 154).

We used the short answer format for the post-test and the delayed-test for two reasons. First, answering multiple-choice questions turned out to be too easy in post-tests, as our pilots revealed (i.e., ceiling effect). Second, answering multiple-choice questions via phone in the delayed test was deemed problematic.

#### Questionnaires and the Phone Interview

In the lab, participants received a pre-questionnaire and two evaluation sheets. The pre-questionnaire included a question on **gender**, two questions on **present mood** (“How are you today?” “Are you looking forward to the following program?” a 6-point smiley scale), and three additional questions irrelevant for present purposes.

The first and the second evaluation sheet included a question on **perceived enjoyment** of the intervention participants just completed (“How did you enjoy the game | quiz?” 6-point smiley scale). These sheets also yielded additional usability data irrelevant for this study (e.g., “What do you think about the number of questions in the game | quiz?” too few—just right—too many).

During the delayed phone interview, we administered the delayed post-test and assessed **domain interest** in the quiz question topics (i.e., interest in space, the human body, nature, and earth geology nature). Domain interest is a trait-like variable connected to voluntary re-engagement with the topic content (Hidi and Renninger, 2006). Accordingly, we examined interest in the topics during leisure time (e.g., “Are you interested in studying space during your leisure time? For instance, do you have a telescope? Do you read books or watch movies about space?”). For each of the four interests, participants received 0, 0.25, 0.5, 0.75, or 1 point (two raters; disagreement resolved by consensus). The resulting interest scores were used only to check whether the four subgroups created by different presentation orders (intervention × question sets) are balanced in the terms of these scores.

#### Intervention—Game

**FIGURE 1:**
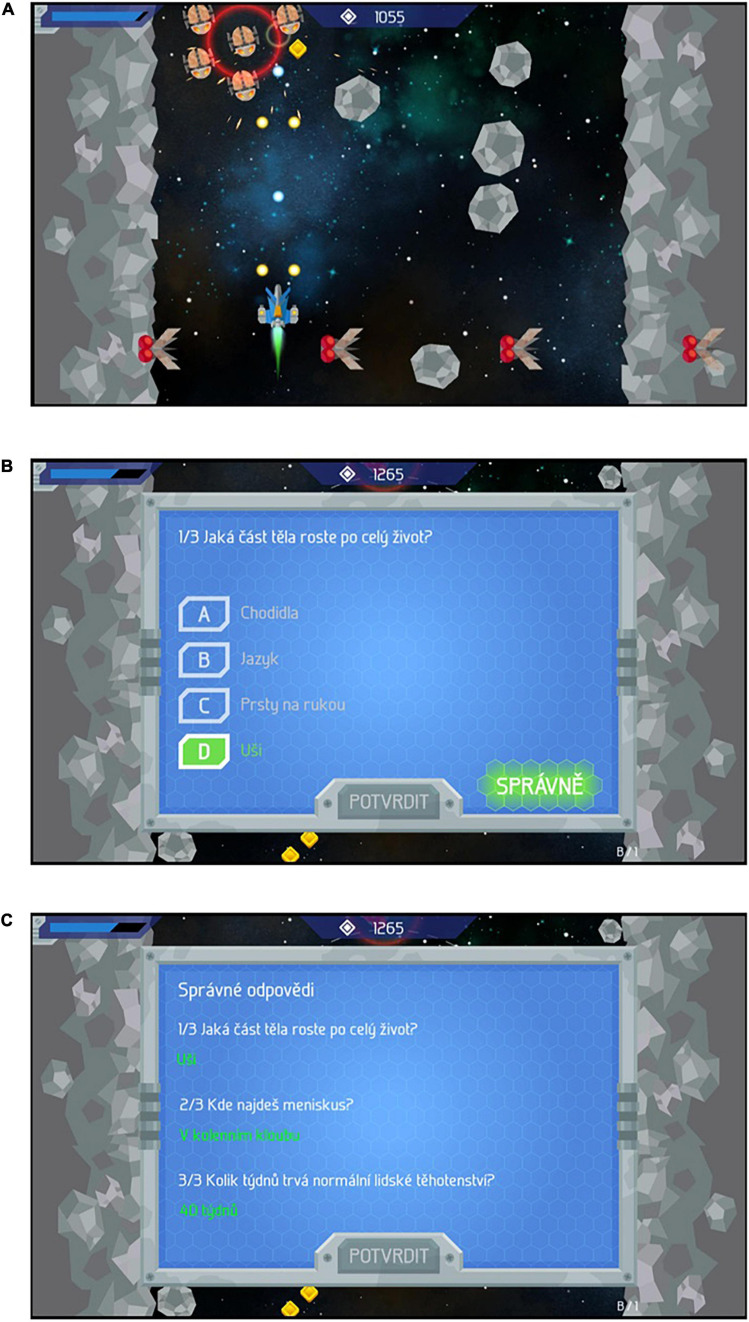
**(A)** Screenshot from the game. The player controls the ship’s movements and he/she can shoot at the space rocks and at alien spaceships moving in the opposite direction. Top left: ship’s remaining “lives.” Top middle: Points. **(B)** Screenshot from the quiz depicting the correct answer; shown after an incorrect response. **(C)** Screenshot from the quiz: all three correct answers are listed after the given trio of questions has been completed. The same graphics are used for the in-game as well as the bare quiz questions. The texts are in the Czech language. [Courtesy of Czech Television (c)].

The game was a single-player 2D bird-eye-view Tyrian-type^1^ space shooter game (Figure 1A). It featured 5 blocks of 1–2-min long gameplay. After each of blocks 1–4, the player’s ship approached a space station and the gameplay was interrupted. The player was presented a sentence superficially wrapping the subsequent quiz questions in a sci-fi narrative, e.g., “Friendly aliens have approached your spaceship and want to ask you a few questions about humans.” Next, the player received three questions from the assigned set of questions (i.e., four trios of questions, one trio after each block). The order of the trios, the presentation order of the three questions within each of the four trios and the order of answer options for each question was randomized. Participants earned points by giving correct answers and could use these points to upgrade their spaceship (e.g., by purchasing weapons, shields). The points were represented as money and the upgrades helped to pass through the block. Otherwise, there was no connection between the gameplay and the questions. When participants answered incorrectly, the correct answer was shown to them (Figure 1B). After each trio, all three correct answers were shown again (Figure 1C). That is, every participant saw the correct answer to each question twice.

#### Intervention—Bare Quiz

The bare quiz showed four trios of questions from the set of questions not used in the game. The questions were displayed as in the game, but the framing narrative and points/money were absent. No gameplay was included.

### Procedure

**FIGURE 2:**
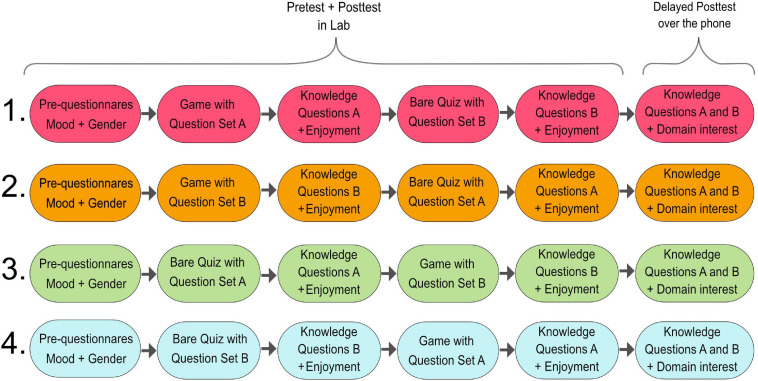
Design of the intervention and procedure.

The study lasted approximately 60 min. After filling in the pre-questionnaire, participants were assigned the order of the interventions (game-first| quiz-first) and the set-order (AB| BA) (Figure 2). Based on this, they started interfacing with the first intervention that featured the appropriate set of questions. After they finished, they received the first evaluation questionnaire and the first post-test that concerned the given set of questions (pen-and-paper). Next, they started interfacing with the second intervention. Thereafter, they received the second evaluation questionnaire and the second post-test that concerned the set of questions assigned second. Finally, participants were offered the option to continue for a few minutes: interfacing either with the game or the bare quiz of their own will (i.e., a free choice motivation period). New questions were used during this period.

The bare quiz interaction was shorter (mean = 5.3, *SD* = 1.4) compared to the quiz (mean = 13.5, *SD* = 1.60). The difference concerned the gameplay.

Approximately 3 weeks later, participants were contacted via phone at a predetermined time. They answered interest questions and, again, 24 test questions. As for the latter questions, the order of the question sets (AB vs. BA) was randomized in the phone testing, but the order of questions within the sets were not. The recorded answers were transcribed for our analysis.

### Data Analysis

We analyzed the data with linear mixed models. For pre-tests and post-tests, the models included a random intercept (participant), fixed factors (intervention type game| bare quiz, question set A| B, and whether the pair of the given intervention and the given question set was assigned first or second; the last variable is called “position”). The position factor addressed potential fatigue/warm-up effects. For delayed tests, the models were similar, but the position corresponded to the position (1^st^/2^nd^) within the phone interview. In all cases, we also included position × intervention interaction to account for potential different initial attitudes toward a game or a bare quiz. The reported models do not involve other interactions; however, both no-interaction and full-interaction models yielded analogous results. For post-tests and delayed tests, we included initial multiple-choice scores as an additional continuous factor. The results of the presented models could be read similarly to ANOVA calculations (American Psychological Association, 2019): we report an omnibus test of each factor or interaction and *p*-values based on degrees of freedom calculated using Satterthwaite’s method (Kuznetsova et al., 2017). We include confidence intervals and Cohen *d* adapted for mixed models (Westfall et al., 2014) as a measure of effect size.

Preference for bare quiz/game was evaluated using a logistic regression. This model initially featured only an intercept; later we tested an extended model which included a game/quiz order.

### Ethics

The experiment was approved as part of a larger project by the Ethics Committee of the Institute of Psychology of the Czech Academy of Sciences. Participants and their legal guardians were informed about the procedure, risks and benefits (though not about the study’s research questions) and could opt to withdraw any time. Legal guardians filled in informed consent forms before the experiment started. The data anonymization was ensured using a numbering system and nick names. Phone interviews were encrypted when moved outside a lab or a home office.

## Results

### Pretests

In pretests, we observed a small difference between question sets [*F*(1, 66) = 4.70, *p* = 0.034, *d* = 0.33]. Children’s scores in Set B were higher by 0.56 (95% CI [0.06, 1.06]; see Table 1 for descriptive data). No other factor yielded significant effect [position, *F*(1, 66) = 0.28, *p* = 0.600; intervention type, *F*(1, 66) = 0.02, *p* = 0.885; position × intervention interaction, *F*(1, 67) = 1.84, *p* = 0.180].

**TABLE 1:** Descriptive statistics of results from pretests, immediate post-tests and delayed tests by intervention type and test position within session.

**Intervention**	**Pretest**		**Post-tests**		**Delayed tests**	
	**Mean**	***SD***	**Mean**	***SD***	**Mean**	***SD***
Game	3.38	1.68	5.93	2.38	4.12	2.15
Game 1^st^	3.51	1.85	5.41	2.37	3.87	2.11
Game 2^nd^	3.24	1.50	6.46	2.30	4.40	2.21
Quiz	3.33	1.80	6.91	2.12	3.96	1.67
Quiz 1^st^	3.03	1.47	6.82	1.94	3.59	1.49
Quiz 2^nd^	3.63	2.04	7.00	2.31	4.29	1.77

### Immediate Post-tests

The performance in post-tests was associated with pretest scores [*F*(1, 109.9) = 5.66, *p* = 0.019]. A difference of one point in a pretest corresponded to an increase by 0.23 points (95% CI [0.01; 0.44]). We observed a significant effect of position [*F*(1, 55.4) = 5.70, *p* = 0.020, *d* = 0.27] and intervention [*F*(1, 55.3) = 16.75, *p* < 0.001, *d* = 0.46]. The tests in the second half of the session yielded higher scores compared to the first half (by 0.58; 95% CI [0.11; 1.04]). Children scored higher when the questions were originally presented in the bare quiz compared to the game (by 0.98; 95% CI [0.52; 1.45]). The remaining effects were not significant [question set version, *F*(1, 57.2) = 0.02, *p* = 0.899; position × intervention, *F*(1, 57.4) = 1.36, *p* = 0.249].

### Delayed Tests

Performance in delayed-tests (*n* = 62) was associated with the original scores in the multiple-choice questions [*F*(1, 117.2) = 20.31, *p* < 0.001]. A difference of one point in pretest corresponded to an increase by 0.41 points (95% CI [0.18; 0.63]). The scores in the second half of the phone call were higher [*F*(1, 45.2) = 4.60, *p* = 0.037, *d* = 0.34]; by 0.59 points (95% CI [0.06; 1.19]). The effect of the intervention was not significant [*F*(1, 45.3) = 0.32, *p* = 0.576; *d* = 0.09], the questions presented during the game yielded higher scores by only 0.15 points (95% CI [-0.38; 0.68]). No other effect was significant [question set version, *F*(1, 47.8) = 0.29, *p* = 0.595; phone-call-position × presentation *F*(1, 46.1) = 0.04, *p* = 0.840]. Because the original session position was significant in post-tests, we later included this parameter in the model. The model fit did not improve [χ^2^(1) = 1.13, *p* = 0.288], the session position was not significant [*F*(1, 44.9) = 1.06, *p* = 0.309], and the remaining results were analogous.

### Enjoyment

Children rated their enjoyment of the game higher by 0.68 point on a 6-point scale (95% CI [0.43, 0.93]; paired *t*-test *t*(68) = 5.39; *p* < 0.001; *d*_*z*_ = 0.65; mean game enjoyment 1.48, *SD* = 0.68), but the bare quiz enjoyment was not low (mean = 2.16, *SD* = 1.04).

### Free-Choice Motivation

When offered a free-choice activity at the end, 59 children opted for the game and 10 for the bare quiz. This preference for the game was significant (odds ratio OR = 5.9; *p* < 0.001; 95% CI [3.16; 12.26]). The position of game/bare quiz did not affect the choice when added to the model (*p* = 0.529).

### Notes on Guessing

In pretests, the responses were very close to the guessing rate of 25% (28.0%, 95% CI [25.8, 30.2]). Open-ended questions used in post-tests and delayed tests did not allow guessing. The children demonstrated a considerable amount of knowledge (post-tests: mean = 6.4, *SD* = 2.3) even after several weeks (delayed tests: mean = 4.0, *SD* = 1.9).

## Discussion

We showed that answering in-game quiz questions results in learning outcomes comparable to answering bare quiz questions, as measured by delayed knowledge tests. In terms of the immediate post-tests, learning from a bare quiz is modestly superior to learning from a quiz within a game. However, this finding is of lesser importance, as long-term retention of knowledge is the key desired educational outcome. We also showed that children enjoyed the game more than the quiz and preferred it in the free-choice period; yet, the bare quiz was also somewhat enjoyed.

From a practical perspective, our findings first and foremost demonstrate that merely answering multiple-choice questions, while obtaining feedback including the correct answers, is sufficient for retaining information over roughly 3 weeks for about 1/3 of questions. This is notable, as unlike in the case of testing effect (Rowland, 2014; Adesope et al., 2017) or clicker-based systems (Chien et al., 2016; Hunsu et al., 2016), we included no additional instructions in the intervention. In this study, and before the delayed test, each question was merely answered twice: during the intervention and in the post-test. Feedback was provided twice during the intervention.

Second, our findings imply that both a quiz within a game and a bare quiz have their place at the table for useful educational interventions. A game with a quiz especially appears to be useful for learning during leisure time periods because children prefer the game and thus they may interface with it longer compared to a bare quiz. A bare quiz appears to be more useful in schooling contexts, where children are *required* to interface with a specific instructional application (i.e., they have no other option). Its advantage is shorter instructional time and cheaper development. Plus, even if it is not preferred, it is also not disliked.

The study’s results are important also theoretically. First, the findings cannot be explained solely within theories that stress the detrimental effects of seductive details (such as cognitive load theory, Sweller et al., 2011; or cognitive theory of multimedia learning, Mayer, 2009). Second, the findings are not consistent with the idea that extrinsic integration of learning content within a game, i.e., a chocolate-covered broccoli approach, is necessarily detrimental to learning. Basically, playing the game, viewed as one large, extrinsically added seductive detail, did not hamper learning outcomes in the long term.

Third, because of null results with regard to learning outcomes, the findings neither support nor refute the behaviorist idea that playing a game as a reward is a good thing. Finally, our findings are consistent with the view that the game was somewhat distractive to learning (as cognitive learning theories and game-based learning literature would predict), *but* higher motivation to engage with in-game quizzes (apparent in our data) counterbalanced this distraction effect. This view is in line with more recent theories of multimedia learning that incorporate motivation into learning processes (see Moreno, 2005; Plass and Kaplan, 2016; Brom et al., 2018; Plass and Kalyuga, 2019). Higher motivation, measured in the game condition, was not of an intrinsic nature but of an extrinsic one (Ryan and Deci, 2000). It could be derived from the money earned for correct answers (that could be spent on the spaceship’s improvements) or simply from the desire to return to the game after the quiz.

It is worth noting that students performed, on average, better in the second post-test and the second delayed test compared to the variants of these tests administered first. We believe this was because children knew better what to expect from the second test after completing the first one (be it the immediate or the delayed test). They could also possibly be more activated when answering the second test. Anyway, this appeared to be a short-term effect, as the effect of the position in the original session was not significant in the delayed test.

### Limitations and Future Directions

The findings have the implications mentioned above, but some caution is needed as concerns generalization. First, we advise against generalizing outside the investigated age groups (children 10–12 years of age). Due to the recruitment procedure, the sample was also skewed toward participants with average or above average socio-economic backgrounds. Older or younger participants, and also children from lower socio-economic backgrounds, may have different attitudes to learning games and quizzes.

Second, some types of game-quiz integration are more meaningful than others. We implemented one specific type of extrinsic game-quiz integration: the meaning of the quiz for the game was provided just by a simple cover story and monetary reward for the spaceship’s upgrades affecting the gameplay. Other methods for how to extrinsically integrate a quiz with a game do exist. In addition, a quiz can be integrated intrinsically, i.e., such that it would not be an interruption of the gameplay but an integral part of it. Plus, the mechanics of a quiz can themselves be gamified; e.g., the process of selecting the answer can be done by means of navigating the player’s avatar through a board-shaped virtual world (cf. Kocadere and Çağlar, 2015). Our results may not necessarily hold for all these methods.

Third, we used single-learner intervention. Collaborative quizzes enable the implementation of competitive and collaborative mechanics, which can alter results. This is because the latter have additional advantages and disadvantages (cf., e.g., Garcia-Sanjuan et al., 2018; Wang and Tahir, 2020).

Fourth, it is not clear whether our results would hold when doing a substantially longer intervention. Fatigue can influence motivation and the ability to learn differently in the game vs. bare quiz.

Finally, we used specific quiz content: factual and conceptual questions on natural sciences topics. It is of interest to find out how in-game vs. bare quizzes would fare when using different content type (e.g., solving mathematical equations), because the content type can influence liking. Our bare quiz was not disliked: it was “somewhat palatable broccoli.” However, was that because of the bare quiz mechanism or due to the learning content type? Could it be that the palatability of solving mathematical questions in a multiple-choice format would differ from our case?

We do not think that these limitations undermine the study’s key findings. Rather, they show directions for future research.

## Conclusion

We tested the instructional efficiency of integrating instructional quizzes within a space shooter game by means of a simple story and a game-relevant reward for correct answers. This straightforward extrinsic integration was neither detrimental nor beneficial to learning in the long term for 10–12-years-old compared to bare instructional quizzes. This suggests that both a quiz extrinsically embedded within a game and a bare quiz are useful tools for knowledge acquisition, albeit for different contexts (leisure time vs. school time). Additional research is needed to elucidate the effects of differently integrated instructional quizzes and what those effects are for different age groups.

## Data Availability Statement

The anonymized datasets for this study can be found in the Open Science Framework repository (https://osf.io/psmkc/).

## Ethics Statement

The experiment was approved as part of a larger project by the Ethics Committee of the Institute of Psychology of the Czech Academy of Sciences. Written informed consent to participate in this study was provided by the participants’ legal guardian/next of kin.

## Author Contributions

CB supervised the whole study and wrote the first draft of the manuscript, except for “Results” section. LJ and CB adapted pen-and-paper materials for this experiment and conducted the pilots, with the help of KV. LJ organized data collection and digitalization. LJ, PS, LK, and TT collected the data. PS also helped with digitalization. KV organized recruitment of participants and development of the intervention. JL run the statistical analyses and wrote “Results” section of the manuscript. CB and LK created the figures. All authors helped to design the study and commented on the manuscript and interpretation of the findings.

## Conflict of Interest

KV declares a potential conflict of interest, as she was employed by Czech Television, which is a public institution engaged (also) in development of educational games. The remaining authors declare the absence of any commercial or financial relationships that could be construed as a potential conflict of interest.

## Publisher’s Note

All claims expressed in this article are solely those of the authors and do not necessarily represent those of their affiliated organizations, or those of the publisher, the editors and the reviewers. Any product that may be evaluated in this article, or claim that may be made by its manufacturer, is not guaranteed or endorsed by the publisher.

## Footnotes

1 https://en.wikipedia.org/wiki/Tyrian_(video_game) (cited 28 February, 2021).
