# Supplementary material for: Extrinsically Integrated Instructional Quizzes in Learning Games: An Educational Disaster or Not?
Source: Front Psychol. 2021 Aug 19;12:678380. doi: 10.3389/fpsyg.2021.678380 (PMC8417244; doi:10.3389/fpsyg.2021.678380)
Supplement: Supplementary file 1 [file Table_1.DOCX]

Supplementary Material A

# Baseline differences

In the reported experiment, participants differed in the quiz/game order condition. We checked the differences between both groups with *t*-tests. We found no differences in initial mood or topic interests.

Table 1. Group differences in mood and interest.

| variable | range | *Mean_GQ_* | *SD_GQ_* | *Mean_QG_* | *SD_QG_* | | Cohen’s *d* | *p* |
| --- | --- | --- | --- | --- | --- | --- | --- | --- |
| Present mood 1^a^ | 1 - 6 | 1.57 | 0.70 | 1.65 | | 0.92 | 0.09 | 0.702 |
| Present mood 2^b^ | 1 - 6 | 1.43 | 0.78 | 1.32 | | 0.68 | 0.14 | 0.553 |
| Interest: space | 0 - 1 | 0.36 | 0.38 | 0.34 | | 0.39 | 0.05 | 0.849 |
| Interest: body | 0 - 1 | 0.15 | 0.26 | 0.16 | | 0.22 | 0.02 | 0.952 |
| Interest: nature | 0 - 1 | 0.42 | 0.36 | 0.41 | | 0.32 | 0.03 | 0.904 |
| Interest: earth geology | 0 - 1 | 0.25 | 0.34 | 0.14 | | 0.29 | 0.35 | 0.167 |

*Notes: GQ and QG correspond to the order of game and quiz during session (GQ is game first, quiz second). ^a^How are you today? (1 – best grade, 6 – worst grade), ^b^Are you looking forward to the following program? (1 – best grade, 6 – worst grade).*

Table 2. Number of children per each game/quiz and question pack order condition.

| order of the game/quiz | order of questions | *N* | *N_boys_* | *N_girls_* |
| --- | --- | --- | --- | --- |
| GQ | AB | 18 | 9 | 9 |
| GQ | BA | 17 | 9 | 8 |
| QG | AB | 18 | 10 | 8 |
| QG | BA | 16 | 8 | 8 |
